# Condition index monitoring supports conservation priorities for the protection of threatened grass-finch populations

**DOI:** 10.1093/conphys/cov025

**Published:** 2015-06-16

**Authors:** Kimberly Maute, Kristine French, Sarah Legge, Lee Astheimer, Stephen Garnett

**Affiliations:** 1 School of Biological Sciences, University of Wollongong, Wollongong, NSW, Australia; 2 Australian Wildlife Conservancy, Mornington Wildlife Sanctuary, PMB 925, Derby, WA, Australia; 4 Deakin University, Geelong, VIC, Australia; 3 Research Institute for the Environment and Livelihood, Charles Darwin University, Casuarina, NT, Australia

**Keywords:** Condition, corticosterone, fat, haematocrit, muscle, season

## Abstract

Conservation agencies are often faced with the difficult task of prioritizing what recovery actions receive support. With the number of species under threat of decline growing globally, research that informs conservation priorities is greatly needed. The relative vulnerability of cryptic or nomadic species is often uncertain, because populations are difficult to monitor and local populations often seem stable in the short term. This uncertainty can lead to inaction when populations are in need of protection. We tested the feasibility of using differences in condition indices as an indication of population vulnerability to decline for related threatened Australian finch sub-species. The Gouldian finch represents a relatively well-studied endangered species, which has a seasonal and site-specific pattern of condition index variation that differs from the closely related non-declining long-tailed finch. We used Gouldian and long-tailed finch condition variation as a model to compare with lesser studied, threatened star and black-throated finches. We compared body condition (fat and muscle scores), haematocrit and stress levels (corticosterone) among populations, seasons and years to determine whether lesser studied finch populations matched the model of an endangered species or a non-declining species. While vulnerable finch populations often had lower muscle and higher fat and corticosterone concentrations during moult (seasonal pattern similar to Gouldian finches), haematocrit values did not differ among populations in a predictable way. Star and black-throated finch populations, which were predicted to be vulnerable to decline, showed evidence of poor condition during moult, supporting their status as vulnerable. Our findings highlight how measures of condition can provide insight into the relative vulnerability of animal and plant populations to decline and will allow the prioritization of efforts towards the populations most likely to be in jeopardy of extinction.

## Teaser

Condition variation in endangered Gouldian and non-declining long-tailed finches was compared to populations of lesser-studied star and black-throated finches. Populations predicted to be vulnerable had patterns of muscle, fat and CORT levels similar to Gouldian finches, suggesting they are at a higher risk of future declines.

## Introduction

Today's conservationists are often faced with the difficult task of promoting species recovery plans to a wide variety of stakeholders in order to fund conservation programmes (Bayon and Jenkins, 2010). Often, key information on the status and ecology of rare species is difficult to obtain or incomplete, leading to uncertainty about the direction and extent of possible recovery actions (Bottril *et al*., 2009). Owing to the number of species and populations potentially under threat of further declines, research and monitoring that informs conservation priorities is greatly needed to support the decision-making process and promote recovery action funding (Marris, 2007; McCarthy *et al*., 2012). For example, many of Australia's granivorous birds have declined rapidly in the past century, with large range contractions documented in half of the native grass-finches in tropical eastern Australia (Franklin, 1999). The relative vulnerability of different granivorous species is often uncertain, because many populations are infrequently monitored, difficult to track due to nomadic behaviour or seem to be stable over the past decade (Garnett *et al*., 2011). The uncertainty regarding the response of populations to management or environmental factors can lead to poor management decisions, and knowledge that assists in the prediction of population responses to change can aid significantly in the decision-making process (Regan *et al*., 2005).

Few actions have been implemented towards granivore recovery outside of conservation reserves, despite a general consensus among ecologists that cascading effects of grazing and increased fire frequency in northern Australian savanna have caused recent declines in granivorous birds (Franklin *et al*., 2005; Garnett *et al*., 2011). Unlike most grass-finches, Gouldian finches (*Erythrura gouldiae*) appear to be particularly sensitive to environmental change due to their specialized grass-seed diet and relatively rigid breeding and moulting schedules (Tidemann and Woinarski, 1994; Dostine and Franklin, 2002). This sensitivity is reflected not only in the disappearance of the species from more than half of its eastern range, but also in the unique seasonal pattern of condition index variation compared with co-occurring finch species that have not declined. Gouldian finches have lower measures of body condition and higher fat scores, haematocrit and avian stress hormone (corticosterone; CORT) values during moult than the breeding season (Maute *et al*., 2013). Non-declining long-tailed (*Poephila acuticauda*) and masked finches (*P. personata*) found in the same habitat did not display these changes in condition in response to seasonal, yearly or site-specific environmental change. Instead, these species tended to be in good body condition year round and had higher CORT levels when breeding compared with moult, as reported for many northern hemisphere passerines (Breuner and Orchinik, 2002; Romero, 2002). The pattern of condition index variation observed in Gouldian finches may be unique or it may be representative of other threatened species that are also sensitive to recent changes in savanna management. If the latter is true, the monitoring of seasonal changes in condition measures could be developed into an important tool for determining the relative vulnerability of populations to further environmental change.

Previous studies have often used the comparison of condition among populations to determine the possible impact of factors such as human disturbance, habitat change or even pollutant exposure (Artacho *et al*., 2007; Ellis *et al*., 2012). Much of this research does support the hypotheses proposed, suggesting that condition indices can be used to determine the responses of individuals and populations to environmental conditions (Dantzer *et al*., 2014). Measures of fat or muscle nutrient storage are easily assessed in a range of plants and animals, and represent an important tool for discovering the relative responses of organisms to environmental change (Stevenson and Woods, 2006). Birds experiencing unpredictable food access show higher levels of fat storage than when food is more accessible, suggesting that fat measures could be used to indicate resource availability levels for bird populations (Ekman and Hake, 1990; Cuthill *et al*., 2000; Cresswell, 2003). Blood measures, such as haematocrit (the proportion of red blood cells to whole blood volume), can indicate the general health of birds, because low levels indicate infection or dehydration, while high levels are often associated with increased exercise, such as during migration (Fair *et al*., 2007). Physiological research investigating levels of stress hormones in animals and heat shock protein levels in plants continues to show variation in the stress levels of organisms in response to environmental change (Busch and Hayward, 2009; Al-Whaibi, 2011; Bauer *et al*., 2013; Chankova *et al*., 2013). Organisms increase the release of stress hormones in response to disturbances, which typically induce feeding or ‘fight-or-flight’ responses that should increase survival probability, as long as the response does not become chronic and reduce breeding and feeding abilities (Sturkie, 1986). The normal variation in condition measures in natural populations could represent a baseline to compare with populations facing new stressors. The existence of multiple grass-finch species and populations that are exposed to a variety of possible environmental stressors in northern Australia presents an opportunity for research into the response of closely related species to habitat change or differences.

The near threatened eastern star finches (*Neochmia ruficauda clarescens*) share several characteristics with Gouldian finches, including living in similar habitats, having a moderately specialized diet of small seeds and strict timing of moult during 3 months each year (Todd *et al*., 2003). In contrast, declining southern black-throated finches (*Poephila cincta cincta*) feed on a larger range of seeds and other food, as well as having a more opportunistic moulting strategy compared with Gouldian or star finches (Higgins *et al*., 2006). Star, black-throated and long-tailed finches all have more opportunistic breeding strategies than Gouldian finches, involving lengthening or shortening of the breeding season (Higgins *et al*., 2006). Together, the life-history traits of star and black-throated finches suggest that they should be more resilient to habitat changes than Gouldian finches; however, large declines have been recorded for these species in northeastern Australia (Garnett *et al*., 2011). The decline of star and black-throated finches suggests that the impacts of increased grazing and improper fire regimens are detrimental and lead to the prediction that the remaining populations that exist in areas subjected to low levels of grazing and improper burning are vulnerable to further declines.

Using predictions of the vulnerability of threatened finch populations to further declines, we aimed to test the hypothesis that changes in condition indices among seasons and years differ between vulnerable and less vulnerable species and populations. We compared changes in condition indices among populations of threatened star, black-throated and Gouldian finches and non-declining long-tailed finches to gain insight into whether declines in some populations are linked to similar physiological responses, despite species differences in life history. Not only will this information help to predict how populations will respond to environmental change, but it will also suggest whether the same environmental processes are likely to be responsible for declines reported for finches across northern Australia.

## Materials and methods

### Species and sites

Sites containing the study species were chosen over a range of habitat types, at paired sites for each species (Table 1). Two populations of the endangered Gouldian finch and non-declining long-tailed finch were sampled; Delamere Station (latitude −15° 27′ 11.3″S, longitude 131° 34′ 14.2″E), a pastoral property with moderate to high grazing levels and no fire, and Bradshaw Field Training Area (latitude −15° 36′ 34.0″S, longitude 130° 24′ 32.4″E), a Defence property that is managed for biodiversity conservation with no grazing and little fire. Both Gouldian finch populations were designated as vulnerable, while both long-tailed finch populations were non-vulnerable, based on previous research on these species and populations that suggested the Gouldian populations fluctuate widely and show evidence of poor condition, while long-tailed finch populations are more stable and retain good condition year round (Woinarski and Tidemann, 1992; Garnett and Crowley, 2000; Maute *et al*., 2013).

**Table 1:** Characteristics of finch monitoring sites and predictions of population vulnerability to decline

Species	Property name	Conservation status	Grazing level	Burning regimen	Predicted vulnerability
Gouldian finch	Delamere Station	Endangered	High	No fire	Vulnerable
Gouldian finch	Bradshaw FTA	Endangered	Low	Moderate, patchy	Vulnerable
Long-tailed finch	Delamere Station	Least concern	High	No fire	Not vulnerable
Long-tailed finch	Bradshaw FTA	Least concern	Low	Moderate, patchy	Not vulnerable
Star finch	Rinyirru (Lakefield) NP	Near threatened	Moderate	Not frequent enough	Vulnerable
Star finch	Pormpurraaw	Near threatened	High, patchy	Frequent, patchy	Not vulnerable
Black-throated finch	Laudham Park Station	Threatened	High	Not frequent enough	Vulnerable
Black-throated finch	Rinyirru (Lakefield) NP	Least concern	Low	Moderate, patchy	Not vulnerable

Predictions are based on the subjective assessment of grazing levels and burning regimens as detrimental to each finch species and population. Abbreviations: FTA, field training area; NP, national park.

Near threatened star finches (*N. r. clarescens*) were sampled at two sites; Pormpuraaw, Queensland (latitude −14° 54′ 18.9″S, longitude 141° 37′ 19.2″E), an area of Indigenous-owned land, and Rinyirru (Lakefield) National Park (latitude 14° 37′ 24.7″S, longitude 143° 58′ 12.8″E), a conservation reserve. While Rinyirru is managed for biodiversity values, managers were not successful in excluding fire or grazing from most of the open grassland used by star finches, with both occurring at moderately high intensity; therefore, this population was predicted to be vulnerable to decline. Pormpuraaw contains patches of unburnt dense coastal forest vegetation, but extensive annual hot bushfires, weed infestations and moderate to high grazing levels in surrounding coastal grassland. Pormpuraaw was predicted to be less vulnerable to decline, due to the stable refuge habitat created by the ungrazed and fire-protected forest vegetation patches.

The non-threatened northern sub-species of the black-throated finch (*P. c. atropygialis*) was sampled at Rinyirru National Park in grassy woodland near densely vegetated riparian habitat that sustains moderate grazing and burning levels. In this area of the reserve, almost no cattle were present and managers were successful in implementing patchy burning every 2–6 years, thus we predicted that this population was not vulnerable to decline. Threatened southern black-throated finches (*P. c. cincta*) were sampled at Laudham Park Station (latitude 19° 28′ 35.6″S, longitude 146° 41′ 14.6″E), a pastoral property with high levels of grazing and little fire. We predicted that this population was vulnerable to decline.

### Finch sampling

Birds were passively captured within 4 h of sunrise using mist nets. No recaptured individuals from this or other studies were included in the analysis. Individuals were captured, measured, banded and released during two seasons: the late dry/early wet season, when birds are moulting (from November to early December 2007 and 2008), or the early dry/late breeding season (from May to June 2008 and 2009). During each visit to a study site, a minimum of 15 individuals of each species were captured and measured. In total, 240 finches (*n* = 60 for each species) are included in our analysis.

Blood samples were taken by venipuncture of the left brachial vein, and a volume of 50–140 µl blood was collected into heparinized haematocrit tubes. We kept the blood samples on ice until we measured the haematocrit and removed the plasma, which was frozen at −80°C within 6 h of sampling. We determined fat score by estimating the volume of fat stored in each bird's furculum (the hollow area below the neck and above the sternum; scale of 0–5) and muscle score based on the shape and relative volume of the pectoral muscles (scale of 0–3; Brown, 1996). To reduce the possibility of observer bias, all bird body scores were assessed by one or two highly skilled observers who always worked together and compared body condition estimation methods weekly.

### Blood measures

Haematocrit was measured after spinning blood in haematocrit tubes for 7 min at a force of 16 060***g*** in a Hettich Haematokrit 210 centrifuge. Total CORT was determined from field-collected plasma samples. For each bird, the length of time between net capture and blood sampling was measured to the nearest minute to account for the effect of capture stress on total plasma CORT concentration. Plasma CORT was measured for 5 μl of whole plasma from each bird in an EIA Kit ACETM Competitive Enzyme Immunoassay for corticosterone (Cayman Chemical Co., Ann Arbor, MI, USA). Two standard CORT solutions (one high concentration and one low) were tested along with finch plasma, which had an intra-assay variability of <5% and an interassay variability of <5%.

**Figure 1:**
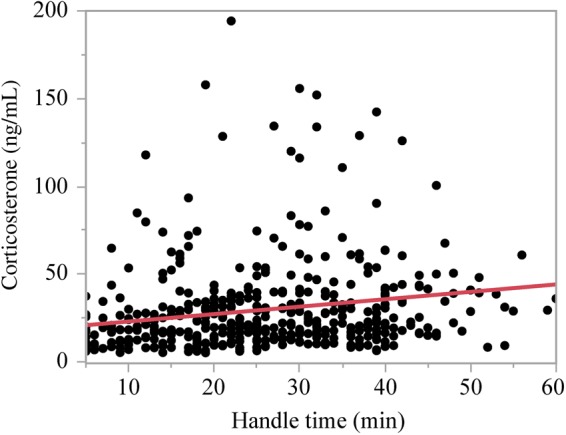
Regression of total corticosterone (in nanograms per millilitre) against handling time (time since capture) using pooled data for all species, seasons, years and sites.

Given that each finch in this study was blood sampled only once over a range of 5–60 min, we created a relative measure of CORT response to handling stress over time, running a linear regression using all CORT results against handling time and using the residuals in subsequent analysis. The regression was significant (*r*^2^ = 0.04 *F*_1,238_ = 23.70, *P* < 0.0001; Fig. 1). Despite the regression accounting for a low level of the variation in total CORT values, we used the residual values from the regression as a measure of stress responses (relative CORT release in response to time since capture) because it removed this low level of handling time variation from subsequent analysis. Residual values (residual CORT) represent the release of CORT corrected for handling time and may be either positive or negative. As the avian stress response is typically non-linear, values near the mean could represent a moderate increase of CORT over time, values below the mean could represent either an attenuation of the stress response or a return to baseline within 60 min, and values above the mean could represent high CORT concentrations released early or retained longer over time. Our aim was to describe differences between populations; therefore, residual CORT values were used as a condition measure in subsequent analysis of the effects of population, season and year (see Maute *et al*., 2013).

### Statistical analysis

For each of the four condition indices, a mixed model restricted maximum likelihood analysis was undertaken, with population type (vulnerable or not vulnerable), year (first year = moult 2007 and breeding 2008; second year = moult 2008 and breeding 2009) and season (breeding vs. moult) designated as fixed factors, and species designated as a random factor to account for general differences in measures among species (SAS institute JMP11.1.1). This ensured that the model was testing for differences in seasonal condition changes among predicted population types. Student's paired *t*-tests were used to identify where differences lay (SAS Institute, JMP11.1.1).

## Results

**Figure 2:**
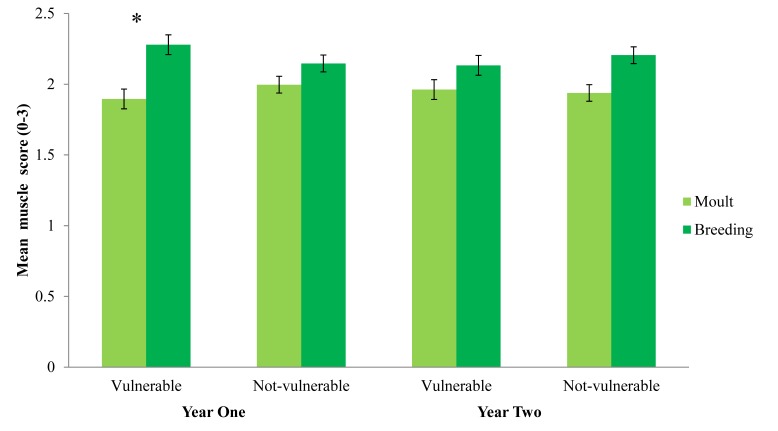
Differences in mean muscle scores among populations (vulnerable or not vulnerable), seasons (moult or breeding) and years (year 1 or year 2). Bars represent means ± SEM. *Significant differences between season values based on Student's paired *t*-tests, *P* < 0.05.

**Figure 3:**
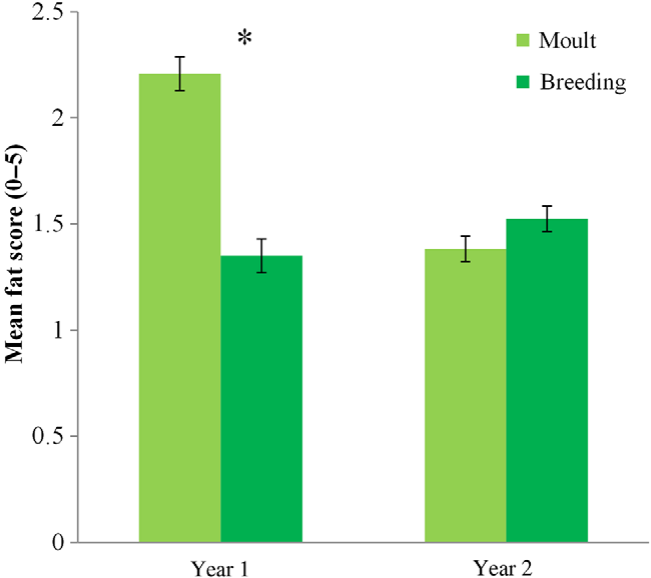
Differences in mean fat scores among seasons (moult or breeding) and years (year 1 or year 2). Bars represent means ± SEM. *Significant differences between season values based on Student's paired *t*-tests, *P* < 0.05.

**Figure 4:**
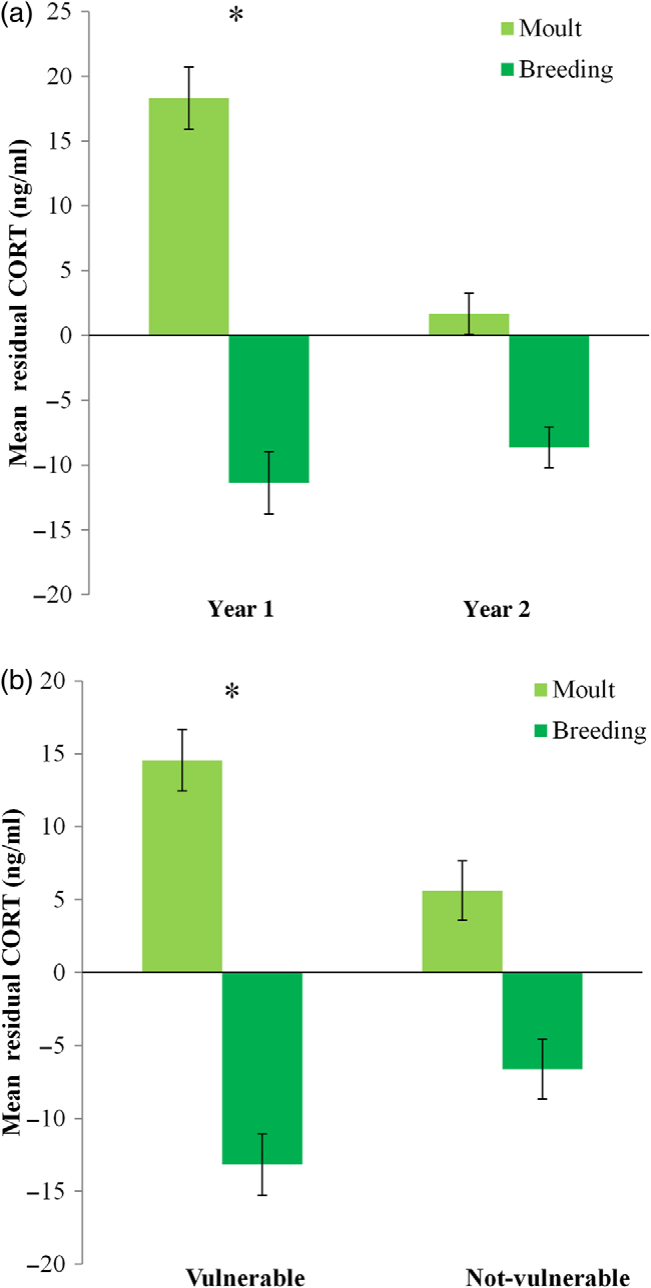
Differences in mean residual corticosterone (CORT; in nanograms per millilitre) among seasons (moult or breeding) during 2 years (**a**) and between vulnerable and not-vulnerable populations (**b**). Bars represent means ± SEM. *Significant differences between seasons based on Student's paired *t*-tests, *P* < 0.05.

Finch condition indices often differed among population types and seasons, and sometimes between years (Table 2). All finch populations had higher muscle scores during breeding compared with moult, and *post hoc* tests revealed that this trend was largely driven by significant differences found in the first year of the study and for vulnerable populations (Table 2 and Fig. 2). Most populations had higher fat scores during moult compared with breeding in year 1, but not in year 2, when fat scores between seasons were similar (Table 2 and Fig. 3). There was a non-significant trend for higher fat scores for vulnerable finch populations (Table 2; vulnerable = 1.70 ± 0.09 SEM, not-vulnerable = 1.53 ± 0.09 SEM). Consistent trends in haematocrit levels were not apparent among seasons, populations or years (Table 2). Residual CORT concentrations were higher during moult compared with breeding for all populations in year 1 but not in year 2 (Table 2 and Fig. 4a). Vulnerable populations had significantly higher residual CORT concentrations during moult than breeding, while not-vulnerable populations did not show a large difference between seasons (Table 2 and Fig. 4b). The mean levels of condition measures for each species are supplied as online supplementary material, because this was not the focus of the study (Supplementary Appendix S1).

**Table 2:** Results of mixed model restricted maximum likelihood analysis evaluating the effects of population vulnerability (vulnerable or not vulnerable), season (breeding or moult) and year (year 1 or year 2) on condition indices (*n* = 240)

Condition index	Muscle score			Fat score		Haematocrit		Residual corticosterone	
Factor(s)	d.f.	*F*-value	*P*-value	*F*-value	*P*-value	*F*-value	*P*-value	*F*-value	*P*-value
Vulnerability	1	0.001	0.97	3.38	0.07	0.52	0.47	0.25	0.62
Season	1	29.12	<0.0001	29.28	<0.0001	1.71	0.19	104.35	<0.0001
Year	1	0.17	0.68	24.08	<0.0001	0.45	0.50	12.65	0.0004
Vulnerability × season	1	0.61	0.43	2.68	0.10	0.12	0.73	15.72	<0.0001
Vulnerability × year	1	0.17	0.68	0.02	0.90	1.07	0.30	0.006	0.94
Season × year	1	0.26	0.61	57.00	<0.0001	1.28	0.26	24.50	<0.0001
Season × year × vulnerability	1	3.24	0.07	1.01	0.31	0.10	0.75	0.81	0.37

Species (Gouldian, long-tailed, star and black-throated finch) was included as a random factor, to account for differences in condition measures among species.

## Discussion

Condition indices are increasingly being promoted as a means to detect vulnerability to environmental change at a population level, particularly for threatened vertebrates (Ellis *et al*., 2012). In the present study, supposed vulnerable threatened star and black-throated finch populations had seasonal variation in condition similar to the uncommon Gouldian finch and unlike the common long-tailed finch. This similarity in condition variation among populations predicted to be vulnerable to decline suggests that the species monitored have similar sensitivity to recent environmental changes that are hypothesized to threaten their long-term persistence. The predictions of population vulnerability used in this study were based on subjective observational assessments by the authors, and similar future studies could be improved if direct environmental measures are employed to create predictions more objectively for a larger number of populations. Despite these shortcomings, our results provide an example of the potential significant insights into conservation questions provided by condition measure monitoring.

### Seasonal variation in stress indices

A striking seasonal change in residual CORT concentrations was observed in all vulnerable populations, with significantly higher CORT during moult than breeding. This differs from the usual pattern of reduced CORT levels found during moult in non-declining long-tailed finch populations and many species in the northern hemisphere (Romero, 2002; Cornelius *et al*., 2012; Maute *et al*., 2013). Though the biological significance and valid interpretation of differences in CORT levels are controversial, the retention of stress responsiveness during moult is likely to imply greater survival potential for finches facing uncertain food resources, because increased CORT has been linked with increased feeding behaviour (Breuner *et al*., 2008, 2013; Schoech *et al*., 2013). Increased CORT concentrations were found in captive black-morph Gouldian finches fed poor diets, and though this did not necessarily reduce their survival or reproductive output, it does suggest a link between nutrient limitations and increased stress in finches (Pryke *et al*., 2012). It is very likely that the grass seeds that wild finches rely on for food are in shorter supply than they have been historically during the late dry/early wet season, which coincides with moult (Andrew and Mott, 1983; Crowley and Garnett, 1998, 1999, 2001; Letnic, 2004). If vulnerable populations of wild finches are experiencing poor nutrition or reduced access to high-quality food during moult, this is a likely explanation for their seasonal changes in CORT levels, but the changes are not a definite indication that these populations will decline.

Non-declining long-tailed finches and less vulnerable populations of star and black-throated finches did not show dramatically higher CORT levels and lower muscle during moult, suggesting that these populations are likely to be better able to adapt to possible changes in food availability and are less likely to require future conservation management compared with vulnerable populations. This assumes that the decreased stress response of supposed non-vulnerable populations during moult shows that these birds were less stressed during this period, but an alternative explanation would suggest that they were suppressing the stress response due to chronic or long-term stressors, which have been shown to downregulate the release of stress hormones in several species (Romero and Wikelski, 2002; Partecke *et al*., 2006; Cyr *et al*., 2007). However, we propose that it is more likely that non-vulnerable populations were simply less stressed, because their body condition profile matched that of birds in good condition (higher muscle and lower fat scores; discussed further below) and did not show evidence of poor condition.

This study used residual CORT concentrations in analysis not only to account for differences in the general stress response values among species, but also to account for differences in the time since capture for individuals within populations. This analysis only described the broadly different stress responses of populations above or below a mean response and would not be suggested as a method to compare responses among individuals. Future research could be improved by sampling individuals at set times after capture so that handling time is less likely to represent a confounding factor. Similar to recent reviews on the use of hormone measures in conservation studies, we also suggest that body condition and other condition measures are recorded to complement measures of stress response (Schoech *et al*., 2013). These methodological improvements would increase the accuracy and validity of the interpretation of variation in stress responses among groups of organisms.

### Seasonal variation in body condition indices

There was a trend for vulnerable populations to have higher fat scores, and all populations showed significant seasonal condition patterns in the first year of the study that matched the Gouldian finch profile of elevated fat storage during moult. Many non-migratory passerines, including grass-finches, rarely carry much fat (Blem, 1976; Breuer *et al*., 1995; Rozman *et al*., 2003). It is theorized that this is because of the disadvantage that extra weight poses to flight agility, energy demands and predator avoidance (Witter and Cuthill, 1993; Brodin, 2001). Despite this possible disadvantage, several winter resident passerines in the northern hemisphere carry larger fat reserves during times of food limitation, and the high levels of fat storage in long-distance migrants are well recorded (Blem, 1976; Ekman and Hake, 1990; Cuthill *et al*., 2000; Cresswell, 2003). The observation of higher fat storage in finches during the late dry season and stressful moult period could be a symptom of unpredictable food availability, because this pattern fits the general profile of birds experiencing food limitations and is very similar to the seasonal pattern of condition indices previously found in Gouldian finches (Maute *et al*., 2013).

In contrast, finch muscle measures showed an opposite seasonal pattern. Muscle scores were higher during breeding compared with moult for all populations, but this trend seemed to be driven by significant differences during year 1 and for vulnerable populations. Muscle is accumulated and lost more slowly than fat in some birds, and lowered muscle mass could be a sign of poor condition and longer-term nutritional stress (Lindstrom and Piersma, 1993). The finding of lower muscle scores in supposed vulnerable populations supports the hypothesis that populations predicted to be more vulnerable to decline are more likely to show evidence of poor condition. Again, this hypothesis could be tested more rigorously with the addition of more replicated populations to this model.

Our analysis did not detect any significant differences in haematocrit levels among the factors tested. Previous surveys of south Australian birds have also failed to detect seasonal changes in bird haematocrit (Breuer *et al*., 1995). It is possible that subtle differences in this blood condition measure among populations were simply not predicted by these factors, but by other unmeasured factors known to influence haematocrit. Infection, due to disease or parasites, and severe malnutrition are known greatly to reduce haematocrit in birds, whereas increases in activity, such as long-distance flights, are known to increase haematocrit (Carpenter, 1975; Jenni *et al*., 2006; Fair *et al*., 2007). However, population-level measures of infection and activity levels would be difficult to obtain and were beyond the scope of the present study. The lack of haematocrit differences among vulnerable or not-vulnerable finch populations might suggest that this measure was a less robust predictor of bird population condition, yet other studies have detected significant differences among populations and experimental treatments that suggest haematocrit was a useful measure for other species and systems (Sánchez-Guzmán *et al*., 2004; Cuervo *et al*., 2007; Bowers *et al*., 2014).

### Inter-annual patterns in condition indices

Surprisingly, all finch populations showed high fat levls and CORT concentrations in late 2007 (year 1), coinciding with high June rainfall (>20 ml) recorded across all of northern Australia and during the middle of the dry season. Unseasonable rain can disrupt the abundance of grass seeds later in the year by promoting premature and extensive germination or burial of seeds that would otherwise be available to finches (Andrew and Mott, 1983; McIvor and Gardner, 1991; Crowley and Garnett, 1999). Thus, it is likely that a widespread rain event led to a shortfall of preferred seed in late 2007, which in turn would lead to higher CORT responses and increased fat storage among all four species of grass-finches. This highlights the usefulness of condition in describing species responses to unusual climatic events, such as winter rainfall in northern Australia, which is likely to increase in the future and may cause further stress to granivorous birds (Hughes, 2011). Most physiological stress studies monitor populations during breeding, because this period is thought to represent the behaviour or season that is most likely to limit population numbers through increased recruitment of young or decreased survival of adults (Dantzer *et al*., 2014). The measurement of condition during multiple life-history periods aided in our discovery that poor food availability during the moult period is just as likely to limit finch populations. Similar stress physiology and body condition comparisons among populations and seasons in other vertebrates and plants could be made to determine the relative risk of population decline due to habitat and climate change. If interpreted cautiously and in association with multiple condition indices, these measures represent innovative and time-effective tools that could be used to inform conservation management decisions.

In conclusion, it seems that not all eastern finches are as sensitive to environmental factors as Gouldian finches; we could infer that this is why the supposed non-vulnerable populations are persisting in the landscape while Gouldian finches have not. However, there are sufficient similarities in the pattern of seasonal variation in these measures for all species to identify food limitation as being a likely cause of their lowered condition indices. The reduction in muscle scores and increase in fat levels and CORT concentrations seen in all populations in late 2007 suggests that all finches responded poorly to a regional change in environmental conditions. In order to buffer remaining populations from similar unforseen detrimental circumstances, care could be taken to minimize nutritional stress during moult in the late dry/early wet season by increasing grass seed production in the remaining habitats of threatened species.

Our findings highlight how measures of condition can provide insight into the relative vulnerability of vertebrate populations to decline and will allow recovery teams to allocate efforts to the populations most likely to be in danger of extirpation. Traditionally, gaining this insight would take many years of costly long-term population monitoring to complete. Our results provide evidence that short-term condition index studies may represent a useful alternative to determining the level of vulnerability among populations when long-term research is not feasible.

## Supplementary material

Supplementary material is available at *Conservation Physiology* online.

## Funding

This work was supported by the Australian Wildlife Conservancy, the Australian Research Council (grant number LP0668122), the University of Wollongong and the Northern Territory Department of Infrastructure, Planning and Environment and was administered and supported by Charles Darwin University. Additional funds were provided by the Stuart Leslie Bird Research fund and the Professor Allen Keast Award (Birdlife Australia).

## Supplementary Material

Supplementary Data
