# Corrigendum to: Condition index monitoring supports conservation priorities for the protection of threatened grass-finch populations

**DOI:** 10.1093/conphys/coy028

**Published:** 2018-06-08

**Authors:** Kimberly Maute, Kristine French, Sarah Legge, Lee Astheimer, Stephen Garnett

**Affiliations:** 1 School of Biological Sciences, University of Wollongong, Wollongong, NSW, Australia; 2 Australian Wildlife Conservancy, Mornington Wildlife Sanctuary, PMB 925, Derby, WA, Australia; 3 Deakin University, Geelong, VIC, Australia; 4 Research Institute for the Environment and Livelihood, Charles Darwin University, Casuarina, NT, Australia

*Conserv Physiol* 3(1): cov025; doi: 10.1093/conphys/cov025

The original version of this manuscript was published with an incorrect figure in the place of figure 2. The manuscript has now been corrected.

