# Supplementary material for: Condition index monitoring supports conservation priorities for the protection of threatened grass-finch populations
Source: Conserv Physiol. 2015 Jun 16;3(1):cov025. doi: 10.1093/conphys/cov025 (PMC4778451; doi:10.1093/conphys/cov025)
Supplement: Supplementary Data [file cov025supp.zip › cov025supp.docx]

Appendix 1: Mean ± SE values for condition measures recorded for each species

| **Species** | **muscle score** | **fat score** | **haematocrit** | **residual CORT level** |
| --- | --- | --- | --- | --- |
| black-throated finch | 2.18 ± 0.04 | 1.68 ± 0.06 | 58.85 ± 0.42 | 5.96 ± 1.89 |
| Gouldian finch | 1.87 ± 0.05 | 1.55 ± 0.07 | 61.09 ± 0.50 | -5.42 ± 2.24 |
| long-tailed finch | 2.02 ± 0.05 | 1.49 ± 0.07 | 59.36 ± 0.50 | -4.65 ± 2.24 |
| star finch | 2.21 ± 0.04 | 1.75 ± 0.06 | 57.10 ± 0.42 | 4.10 ± 1.89 |
